# Phenyl *N*-(5-chloro-2-nitro­phenyl)carbamate

**DOI:** 10.1107/S1600536812030930

**Published:** 2012-07-25

**Authors:** Bao-Hua Zou, Zheng Fang, Hui Zhong, Guo Kai, Ping Wei

**Affiliations:** a School of Pharmaceutical Sciences, Nanjing University of Technology, Puzhunan Road No. 30 Nanjing, Nanjing 210009, People’s Republic of China; b College of Life Science and Pharmaceutical Engineering, Nanjing University of Technology, Puzhunan Road No. 5 Nanjing, Nanjing 210009, People’s Republic of China

## Abstract

In the title compound, C_13_H_9_ClN_2_O_4_, the dihedral angle between the benzene rings is 79.5 (1)°. The mean plane of the carbamate group makes angles of 7.4 (2) and 73.6 (2)° with the mean planes of the two benzene rings. In the crystal, weak C—H⋯O inter­actions are observed between the mol­ecules, connecting them into a two-dimensional network.

## Related literature

For details of dovitinib, of which the title compound is a derivative, see: Huynh (2010 ▶). For the synthesis of the title compound, see: Bandgar *et al.* (2011 ▶). For bond lengths, see: Zhu *et al.* (2007 ▶).
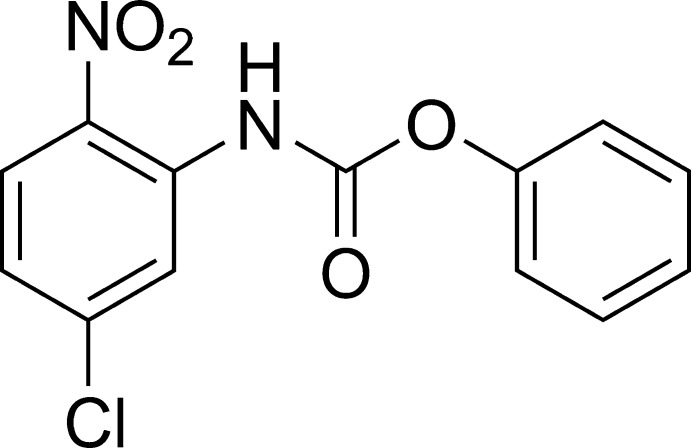


## Experimental

### 

#### Crystal data

C_13_H_9_ClN_2_O_4_
*M* *_r_* = 292.67
Monoclinic, 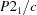

*a* = 8.4760 (17) Å
*b* = 5.9270 (12) Å
*c* = 24.996 (5) Å
β = 94.77 (3)°
*V* = 1251.4 (4) Å^3^
*Z* = 4
Mo *K*α radiation
μ = 0.32 mm^−1^
*T* = 293 K
0.30 × 0.20 × 0.10 mm

#### Data collection

Enraf–Nonius CAD-4 diffractometer
Absorption correction: ψ scan (North *et al.*, 1968 ▶) *T*_min_ = 0.910, *T*_max_ = 0.969
2466 measured reflections
2300 independent reflections
1593 reflections with *I* > 2σ(*I*)
*R*_int_ = 0.084
3 standard reflections every 200 reflections intensity decay: 1%

#### Refinement

*R*[*F* ^2^ > 2σ(*F* ^2^)] = 0.046
*wR*(*F* ^2^) = 0.155
*S* = 1.00
2300 reflections
182 parameters
H-atom parameters constrained
Δρ_max_ = 0.22 e Å^−3^
Δρ_min_ = −0.22 e Å^−3^

### 

Data collection: *CAD-4 EXPRESS* (Enraf–Nonius, 1994 ▶); cell refinement: *CAD-4 EXPRESS*; data reduction: *XCAD4* (Harms & Wocadlo, 1995 ▶); program(s) used to solve structure: *SHELXS97* (Sheldrick, 2008 ▶); program(s) used to refine structure: *SHELXL97* (Sheldrick, 2008 ▶); molecular graphics: *SHELXTL-Plus* (Sheldrick, 2008 ▶); software used to prepare material for publication: *SHELXL97*.

## Supplementary Material

Crystal structure: contains datablock(s) global, I. DOI: 10.1107/S1600536812030930/jj2143sup1.cif

Structure factors: contains datablock(s) I. DOI: 10.1107/S1600536812030930/jj2143Isup2.hkl

Supplementary material file. DOI: 10.1107/S1600536812030930/jj2143Isup3.cml

Additional supplementary materials:  crystallographic information; 3D view; checkCIF report

## Figures and Tables

**Table 1:** Hydrogen-bond geometry (Å, °)

*D*—H⋯*A*	*D*—H	H⋯*A*	*D*⋯*A*	*D*—H⋯*A*
C4—H4*A*⋯O2^i^	0.93	2.48	3.312 (4)	150
C13—H13*A*⋯O1^ii^	0.93	2.56	3.419 (4)	154

Symmetry codes: (i) 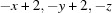; (ii) 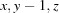.

**Fig. 1.**
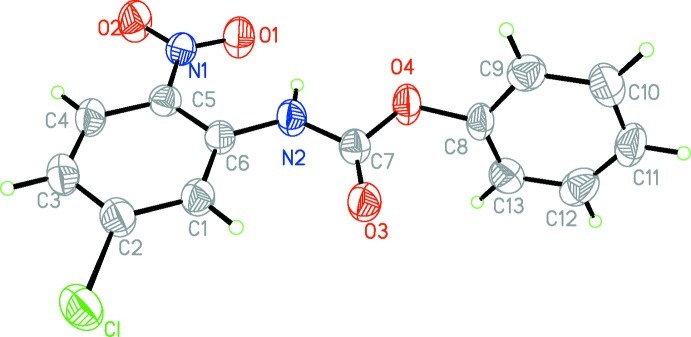
The molecular structure of the title compound, (I), showing the atom-labeling scheme and 50% probability displacement ellipsoids.

**Fig. 2.**
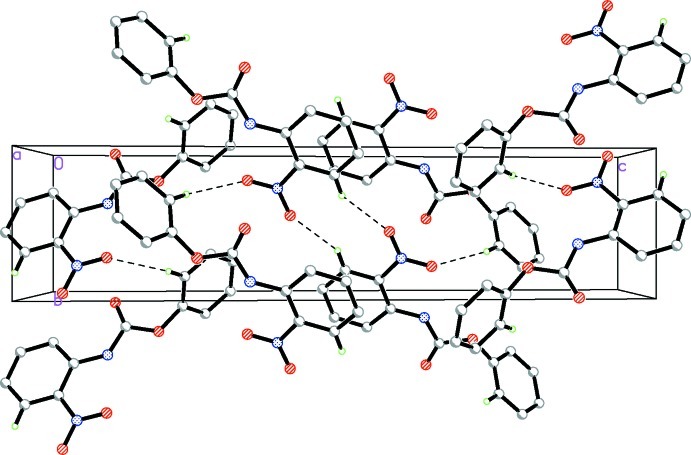
Packing diagram of the title compound viewed along the a axis. Dashed lines indicate weak C—H···O intermolecular interactions which link the molecules into a two-dimensional network array. Remaining H atoms have been omitted for clarity.

## supplementary crystallographic information

### Comment

The title compound, C_13_H_9_ClN_2_O_4_, (I), is an important derivative of Dovitinib (Huynh, 2010). We report herein its crystal structure.

In the title compound, C_13_H_9_ClN_2_O_4_, the dihedral angle between the two benzene rings is 79.5 (1)° (Fig. 1). The angles between the mean plane of the carbamate group (N2/C7/O3/O4) and the two 6-membered benzene rings (C1–C6 and C8–C13) is 7.4° and 73.6°, respectively. Bond lengths are in normal ranges (Zhu *et al.*, 2007). In the crystal structure, weak C—H···O (Table 1 )intermolecular interactions are observed which link the molecules into a two-dimensional network array (Fig. 2).

### Experimental

5-chloro-2-nitroaniline (10.46 mmol, 1.80 g) and Et3N (1.5 ml) were dissolved in dichloromethane (30 ml). Phenyl carbonochloridate (19.23 mmol, 3.01 g) was added to the solution and the reaction mixture stired at room temperature for 5 h. The solution was washed with water (15 ml) for 3 times, dried and concentrated to get the crude. The crude was purified by ethanol to get the title compound (1.83 g) (Bandgar *et al.* 2011). pure: yellow solid. Crystals of the title compound for X-ray diffraction were obtained by slow evaporation of an ethanol solution.

### Refinement

H atoms were positioned geometrically with C—H = 0.93 and N—H = 0.86 for aromatic and amine, respectively, and constrained to ride on their parent atoms, with *U*_iso_(H) = 1.2 times *U*_eq_(C).

### Crystal data

**Table:**

C_13_H_9_ClN_2_O_4_	*F*(000) = 600
*M**_r_* = 292.67	*D*_x_ = 1.553 Mg m^−^^3^
Monoclinic, *P*2_1_/*c*	Mo *K*α radiation, λ = 0.71073 Å
Hall symbol: -P 2ybc	Cell parameters from 25 reflections
*a* = 8.4760 (17) Å	θ = 9–13°
*b* = 5.9270 (12) Å	µ = 0.32 mm^−^^1^
*c* = 24.996 (5) Å	*T* = 293 K
β = 94.77 (3)°	Block, yellow
*V* = 1251.4 (4) Å^3^	0.30 × 0.20 × 0.10 mm
*Z* = 4	

### Data collection

**Table:**

Enraf–Nonius CAD-4 diffractometer	1593 reflections with *I* > 2σ(*I*)
Radiation source: fine-focus sealed tube	*R*_int_ = 0.084
Graphite monochromator	θ_max_ = 25.4°, θ_min_ = 1.6°
ω/2θ scans	*h* = 0→10
Absorption correction: ψ scan (North *et al.*, 1968)	*k* = 0→7
*T*_min_ = 0.910, *T*_max_ = 0.969	*l* = −30→30
2466 measured reflections	3 standard reflections every 200 reflections
2300 independent reflections	intensity decay: 1%

### Refinement

**Table:**

Refinement on *F*^2^	Secondary atom site location: difference Fourier map
Least-squares matrix: full	Hydrogen site location: inferred from neighbouring sites
*R*[*F*^2^ > 2σ(*F*^2^)] = 0.046	H-atom parameters constrained
*wR*(*F*^2^) = 0.155	*w* = 1/[σ^2^(*F*_o_^2^) + (0.095*P*)^2^] where *P* = (*F*_o_^2^ + 2*F*_c_^2^)/3
*S* = 1.00	(Δ/σ)_max_ < 0.001
2300 reflections	Δρ_max_ = 0.22 e Å^−^^3^
182 parameters	Δρ_min_ = −0.22 e Å^−^^3^
0 restraints	Extinction correction: *SHELXL97* (Sheldrick, 2008), Fc^*^=kFc[1+0.001xFc^2^λ^3^/sin(2θ)]^-1/4^
Primary atom site location: structure-invariant direct methods	Extinction coefficient: 0.022 (4)

### Special details

**Table:**

Geometry. All e.s.d.'s (except the e.s.d. in the dihedral angle between two l.s. planes) are estimated using the full covariance matrix. The cell e.s.d.'s are taken into account individually in the estimation of e.s.d.'s in distances, angles and torsion angles; correlations between e.s.d.'s in cell parameters are only used when they are defined by crystal symmetry. An approximate (isotropic) treatment of cell e.s.d.'s is used for estimating e.s.d.'s involving l.s. planes.
Refinement. Refinement of *F*^2^ against ALL reflections. The weighted *R*-factor *wR* and goodness of fit *S* are based on *F*^2^, conventional *R*-factors *R* are based on *F*, with *F* set to zero for negative *F*^2^. The threshold expression of *F*^2^ > σ(*F*^2^) is used only for calculating *R*-factors(gt) *etc*. and is not relevant to the choice of reflections for refinement. *R*-factors based on *F*^2^ are statistically about twice as large as those based on *F*, and *R*-factors based on ALL data will be even larger.

### Fractional atomic coordinates and isotropic or equivalent isotropic displacement parameters (Å^2^)

**Table:**

	*x*	*y*	*z*	*U*_iso_*/*U*_eq_	
Cl	0.57503 (11)	0.19442 (15)	−0.05438 (3)	0.0609 (3)	
O1	1.0095 (3)	0.7195 (4)	0.14287 (9)	0.0616 (7)	
N1	0.9731 (3)	0.7720 (4)	0.09605 (10)	0.0423 (6)	
C1	0.7096 (3)	0.2956 (5)	0.04383 (11)	0.0414 (7)	
H1A	0.6625	0.1637	0.0548	0.050*	
O2	1.0200 (3)	0.9469 (4)	0.07710 (9)	0.0662 (7)	
N2	0.8211 (3)	0.3679 (4)	0.13502 (9)	0.0459 (6)	
H2A	0.8800	0.4568	0.1552	0.055*	
C2	0.6891 (3)	0.3616 (5)	−0.00907 (11)	0.0440 (7)	
O3	0.6855 (3)	0.0324 (4)	0.14033 (8)	0.0499 (6)	
C3	0.7548 (4)	0.5578 (5)	−0.02738 (12)	0.0515 (8)	
H3A	0.7379	0.6001	−0.0632	0.062*	
O4	0.7949 (3)	0.2174 (4)	0.21348 (8)	0.0523 (6)	
C4	0.8447 (4)	0.6877 (5)	0.00832 (11)	0.0455 (7)	
H4A	0.8895	0.8203	−0.0033	0.055*	
C5	0.8702 (3)	0.6245 (5)	0.06190 (11)	0.0380 (7)	
C6	0.8009 (3)	0.4261 (5)	0.08095 (11)	0.0370 (6)	
C7	0.7591 (3)	0.1870 (5)	0.16017 (11)	0.0384 (7)	
C8	0.7459 (3)	0.0532 (5)	0.24940 (10)	0.0400 (7)	
C9	0.6295 (4)	0.1140 (5)	0.28161 (12)	0.0458 (7)	
H9A	0.5780	0.2521	0.2771	0.055*	
C10	0.5908 (4)	−0.0359 (6)	0.32108 (12)	0.0546 (9)	
H10A	0.5124	0.0013	0.3434	0.066*	
C11	0.6686 (4)	−0.2403 (6)	0.32724 (12)	0.0563 (9)	
H11A	0.6420	−0.3405	0.3537	0.068*	
C12	0.7838 (4)	−0.2955 (6)	0.29484 (13)	0.0575 (9)	
H12A	0.8357	−0.4334	0.2993	0.069*	
C13	0.8247 (4)	−0.1489 (5)	0.25529 (12)	0.0487 (8)	
H13A	0.9037	−0.1863	0.2332	0.058*	

### Atomic displacement parameters (Å^2^)

**Table:**

	*U*^11^	*U*^22^	*U*^33^	*U*^12^	*U*^13^	*U*^23^
Cl	0.0763 (6)	0.0643 (6)	0.0401 (5)	−0.0168 (4)	−0.0063 (4)	−0.0024 (4)
O1	0.0783 (17)	0.0571 (14)	0.0470 (14)	−0.0243 (12)	−0.0099 (11)	0.0075 (11)
N1	0.0454 (14)	0.0396 (14)	0.0428 (14)	−0.0047 (11)	0.0083 (11)	0.0038 (11)
C1	0.0463 (17)	0.0399 (16)	0.0381 (15)	−0.0066 (13)	0.0039 (12)	0.0038 (13)
O2	0.0900 (18)	0.0517 (14)	0.0564 (15)	−0.0300 (13)	0.0034 (12)	0.0098 (11)
N2	0.0598 (16)	0.0443 (14)	0.0329 (13)	−0.0182 (12)	−0.0011 (11)	0.0030 (11)
C2	0.0477 (18)	0.0470 (17)	0.0368 (16)	−0.0028 (14)	0.0005 (12)	0.0010 (13)
O3	0.0651 (14)	0.0470 (12)	0.0369 (11)	−0.0183 (11)	−0.0002 (9)	0.0031 (9)
C3	0.067 (2)	0.0513 (19)	0.0356 (16)	−0.0035 (16)	0.0020 (14)	0.0082 (14)
O4	0.0728 (15)	0.0521 (13)	0.0311 (11)	−0.0239 (11)	−0.0018 (10)	0.0077 (9)
C4	0.0550 (18)	0.0428 (17)	0.0391 (16)	−0.0037 (14)	0.0059 (13)	0.0097 (13)
C5	0.0383 (15)	0.0365 (15)	0.0394 (15)	−0.0021 (12)	0.0050 (12)	−0.0005 (12)
C6	0.0396 (15)	0.0377 (15)	0.0338 (14)	0.0009 (12)	0.0038 (11)	0.0032 (12)
C7	0.0391 (15)	0.0413 (16)	0.0344 (15)	−0.0010 (13)	0.0010 (12)	0.0036 (13)
C8	0.0473 (17)	0.0441 (17)	0.0276 (14)	−0.0128 (14)	−0.0021 (12)	0.0062 (12)
C9	0.0500 (18)	0.0413 (16)	0.0452 (17)	−0.0027 (14)	−0.0006 (13)	−0.0022 (13)
C10	0.058 (2)	0.067 (2)	0.0403 (17)	−0.0212 (18)	0.0119 (14)	−0.0078 (16)
C11	0.078 (2)	0.054 (2)	0.0351 (16)	−0.0228 (19)	−0.0020 (16)	0.0113 (15)
C12	0.071 (2)	0.0434 (19)	0.055 (2)	−0.0017 (17)	−0.0110 (17)	0.0074 (16)
C13	0.0496 (18)	0.0526 (19)	0.0437 (17)	−0.0007 (15)	0.0033 (13)	−0.0023 (14)

### Geometric parameters (Å, º)

**Table:**

Cl—C2	1.737 (3)	O4—C8	1.410 (3)
O1—N1	1.226 (3)	C4—C5	1.390 (4)
N1—O2	1.220 (3)	C4—H4A	0.9300
N1—C5	1.459 (4)	C5—C6	1.415 (4)
C1—C2	1.376 (4)	C8—C9	1.372 (4)
C1—C6	1.392 (4)	C8—C13	1.374 (4)
C1—H1A	0.9300	C9—C10	1.387 (4)
N2—C7	1.369 (4)	C9—H9A	0.9300
N2—C6	1.391 (3)	C10—C11	1.382 (5)
N2—H2A	0.8600	C10—H10A	0.9300
C2—C3	1.384 (4)	C11—C12	1.360 (5)
O3—C7	1.193 (3)	C11—H11A	0.9300
C3—C4	1.363 (4)	C12—C13	1.382 (4)
C3—H3A	0.9300	C12—H12A	0.9300
O4—C7	1.354 (3)	C13—H13A	0.9300
			
O2—N1—O1	121.5 (3)	N2—C6—C5	120.8 (2)
O2—N1—C5	118.7 (2)	C1—C6—C5	117.4 (2)
O1—N1—C5	119.8 (2)	O3—C7—O4	125.3 (3)
C2—C1—C6	120.1 (3)	O3—C7—N2	128.2 (3)
C2—C1—H1A	119.9	O4—C7—N2	106.6 (2)
C6—C1—H1A	119.9	C9—C8—C13	122.2 (3)
C7—N2—C6	128.3 (2)	C9—C8—O4	117.2 (3)
C7—N2—H2A	115.9	C13—C8—O4	120.3 (3)
C6—N2—H2A	115.9	C8—C9—C10	118.2 (3)
C1—C2—C3	122.3 (3)	C8—C9—H9A	120.9
C1—C2—Cl	118.9 (2)	C10—C9—H9A	120.9
C3—C2—Cl	118.8 (2)	C11—C10—C9	120.2 (3)
C4—C3—C2	118.5 (3)	C11—C10—H10A	119.9
C4—C3—H3A	120.8	C9—C10—H10A	119.9
C2—C3—H3A	120.8	C12—C11—C10	120.3 (3)
C7—O4—C8	118.8 (2)	C12—C11—H11A	119.9
C3—C4—C5	120.8 (3)	C10—C11—H11A	119.9
C3—C4—H4A	119.6	C11—C12—C13	120.7 (3)
C5—C4—H4A	119.6	C11—C12—H12A	119.7
C4—C5—C6	120.8 (3)	C13—C12—H12A	119.7
C4—C5—N1	116.1 (2)	C8—C13—C12	118.5 (3)
C6—C5—N1	123.1 (2)	C8—C13—H13A	120.8
N2—C6—C1	121.8 (2)	C12—C13—H13A	120.8
			
C6—C1—C2—C3	−0.9 (5)	C4—C5—C6—C1	1.3 (4)
C6—C1—C2—Cl	179.8 (2)	N1—C5—C6—C1	−177.7 (3)
C1—C2—C3—C4	1.0 (5)	C8—O4—C7—O3	1.5 (4)
Cl—C2—C3—C4	−179.7 (2)	C8—O4—C7—N2	−179.5 (2)
C2—C3—C4—C5	0.1 (5)	C6—N2—C7—O3	6.6 (5)
C3—C4—C5—C6	−1.3 (5)	C6—N2—C7—O4	−172.3 (3)
C3—C4—C5—N1	177.8 (3)	C7—O4—C8—C9	−111.0 (3)
O2—N1—C5—C4	5.3 (4)	C7—O4—C8—C13	75.8 (3)
O1—N1—C5—C4	−175.2 (3)	C13—C8—C9—C10	−0.4 (4)
O2—N1—C5—C6	−175.6 (3)	O4—C8—C9—C10	−173.4 (2)
O1—N1—C5—C6	3.9 (4)	C8—C9—C10—C11	0.0 (4)
C7—N2—C6—C1	−0.6 (5)	C9—C10—C11—C12	0.3 (5)
C7—N2—C6—C5	178.0 (3)	C10—C11—C12—C13	−0.1 (5)
C2—C1—C6—N2	178.4 (3)	C9—C8—C13—C12	0.5 (4)
C2—C1—C6—C5	−0.2 (4)	O4—C8—C13—C12	173.3 (3)
C4—C5—C6—N2	−177.3 (3)	C11—C12—C13—C8	−0.2 (5)
N1—C5—C6—N2	3.7 (4)		

### Hydrogen-bond geometry (Å, º)

**Table:**

*D*—H···*A*	*D*—H	H···*A*	*D*···*A*	*D*—H···*A*
C4—H4*A*···O2^i^	0.93	2.48	3.312 (4)	150
C13—H13*A*···O1^ii^	0.93	2.56	3.419 (4)	154

Symmetry codes: (i) −*x*+2, −*y*+2, −*z*; (ii) *x*, *y*−1, *z*.
